# Impairment of SK-MEL-28 Development—A Human Melanoma Cell Line—By the *Crataeva tapia* Bark Lectin and Its Sequence-Derived Peptides

**DOI:** 10.3390/ijms241310617

**Published:** 2023-06-25

**Authors:** Kathleen Chwen Ming Lie, Camila Ramalho Bonturi, Bruno Ramos Salu, Juliana Rodrigues de Oliveira, Márcia Bonini Galo, Patrícia Maria Guedes Paiva, Maria Tereza dos Santos Correia, Maria Luiza Vilela Oliva

**Affiliations:** 1 Department of Biochemistry, Universidade Federal de São Paulo, São Paulo 04044-020, Brazil; kalimryn@gmail.com (K.C.M.L.);; 2 Department of Biochemistry, Universidade Federal de Pernambuco, Recife 50670-910, Brazil

**Keywords:** cancer, CrataBL, lectin, melanoma, peptides, protease inhibitors

## Abstract

Melanoma is difficult to treat with chemotherapy, prompting the need for new treatments. Protease inhibitors have emerged as promising candidates as tumor cell proteases promote metastasis. Researchers have developed a chimeric form of the *Bauhinia bauhinioides* kallikrein inhibitor, rBbKIm, which has shown negative effects on prostate tumor cell lines DU145 and PC3. *Crataeva tapia* bark lectin, CrataBL, targets sulfated oligosaccharides in glycosylated proteins and has also demonstrated deleterious effects on prostate and glioblastoma tumor cells. However, neither rBbKIm nor its derived peptides affected the viability of SK-MEL-28, a melanoma cell line, while CrataBL decreased viability by over 60%. Two peptides, Pep. 26 (Ac-Q-N-S-S-L-K-V-V-P-L-NH2) and Pep. 27 (Ac-L-P-V-V-K-L-S-S-N-Q-NH2), were also tested. Pep. 27 suppressed cell migration and induced apoptosis when combined with vemurafenib, while Pep. 26 inhibited cell migration and reduced nitric oxide and the number of viable cells. Vemurafenib, a chemotherapy drug used to treat melanoma, was found to decrease the release of interleukin 8 and PDGF-AB/BB cytokines and potentiated the effects of proteins and peptides in reducing these cytokines. These findings suggest that protease inhibitors may be effective in blocking melanoma cells and highlight the potential of CrataBL and its derived peptides.

## 1. Introduction

Lectins are a class of proteins that reversibly bind to carbohydrates other than enzymes or antibodies. They are found in most living organisms, such as viruses, bacteria, plants, and animals [1], and are involved in numerous cellular processes such as binding to glycoconjugate on the cell surface from circulating proteins [2], targeting of host pathogenic cells [3], recruiting of leukocytes to sites of inflammation [4], and cell interactions with target proteins from the immune system that can then trigger antitumor activity [5]. Plant lectins are found in foods such as lentils, soybeans, tomatoes, peanuts, bananas, mushrooms, corn, potatoes, rice, and wheat. The amount ingested can be significant, because many lectins are resistant to intestinal digestion and enter the circulatory system intact, preserving their complete biological properties [6]. Lectins can be cytotoxic, induce apoptosis, and inhibit tumor growth by binding to carbohydrates moieties of cancer cell membranes or receptors, which are exacerbated in various types of cancer [7].

CrataBL is a lectin extracted from the bark of the *Crataeva tapia* tree, belonging to the Capparaceae family, found in northeastern Brazil, and purified according to the Araújo protocol [8]. It is a 20 kDa Kunitz-type inhibitor as it inhibits trypsin (43 µM) and factor Xa (8.6 µM) [9], binds to sulfated oligosaccharides [10], affects the development of *Callosobruchus maculatus* larvae [11], blocks coagulation and arterial thrombus formation [12], induces apoptosis by releasing mitochondrial cytochrome c and activating caspase-3 in DU145 and PC3 prostate cancer cells [9], and inhibits invasion and adhesion of U87 glioblastoma cells [13].

Another protein with deleterious effects on DU145 and PC3 prostate tumor cell lines [14] is rBbKIm, a recombinant and modified form of *Bauhinia bauhinioides* kallikrein inhibitor that contains the RGD and RGE motifs found in the *Bauhinia rufa* trypsin inhibitor. In addition to being important for integrin signaling, these sequences also play an important role in plant defense [15].

Melanoma is a cutaneous cancer with a higher mortality than other skin tumors because it exhibits an aggressive tumor behavior, and its incidence is increasing worldwide [16]. SK-MEL-28 is a lineage of human melanoma that harbors the BRAF *V600E* mutation [17], which enhances and selects sensitivity to mitogen-activated protein kinase kinase (MEK) inhibition [18] compared to “wild-type” cells, causing deregulation of the proliferation, survival, and metastasis of cancer cells [19]. Vemurafenib has been used since 2011 as an inhibitor of *v-raf murine sarcoma viral oncogene homolog B1 (BRAF)* kinase activity; however, diverse mechanisms of resistance have been identified in melanoma, which often leads to disease progression in patients carrying this mutation [19]. In this study, we investigated the cytotoxic effects of CrataBL, rBbKIm, and their sequence-derived peptides in this cell line. Furthermore, combination treatment with vemurafenib was also evaluated to determine whether the effect would be synergistic and more prominent.

## 2. Results

### 2.1. CrataBL Shows a Deleterious Effect on SK-MEL-28 Cell Viability Compared to rBbKIm and Has Low Toxicity on Healthy Fibroblast Cells

**Figure 1:**
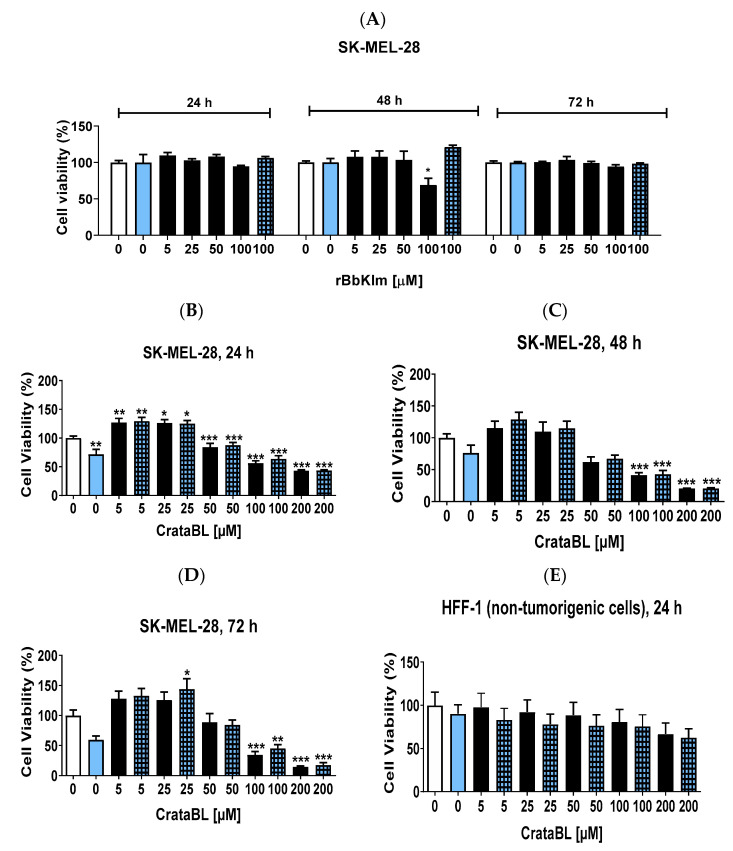

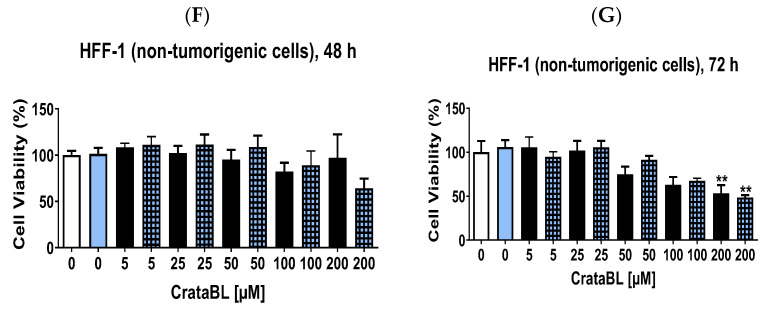
CrataBL potentially affects SK-MEL-28 cell viability compared to recombinant protein rBbKIm and presents low toxicity on healthy fibroblast cells. To better interpret the results of the graphs, the white columns represent the control group (untreated cells), the blue column represents cells treated with 2 µM of vemurafenib alone, the black columns represent cells treated only with the proteins rBbKIm or CrataBL, and the light blue square columns represent the combined treatment (protein and an additional 2 µM of vemurafenib). Treatment with rBbKIm from 5 to 100 µM in the three periods and 100 µM of rBbKIm with 2 µM of vemurafenib (**A**). Concentrations of 5 to 200 µM of CrataBL and the combined therapy in SK-MEL-28 for 24 (**B**), 48 (**C**), and 72 h (**D**). Concentrations of 5 to 200 µM of CrataBL in HFF-1 healthy cells for 24 (**E**), 48 (**F**), and 72 h (**G**). (*) results are statistically different from control (* *p* < 0.05, ** *p* < 0.005, and *** *p* < 0.0005), one-way ANOVA and Tukey test.

The recombinant protein, rBbKIm, was tested in melanoma cells and treatment with a concentration of 100 µM showed a decrease in viability of approximately 20% within 48 h, but this effect was reversed after 72 h. There was no reduction in the viability of cells treated with 100 µM rBbKIm combined with vemurafenib (Figure 1A). The selected concentration of vemurafenib was 2 µM for all cell assays below (Figure S1). Moreover, no reduction in cell viability was observed in cells treated with the peptides containing the RGD and RGE motif alone and in combination with 2 µM of vemurafenib at a higher dose (Figure S2).

The effects of CrataBL on melanoma cell viability are shown in Figure 1C–E. At concentrations of 200 µM, the inhibition rate was higher than 50% in the first 24 h of treatment (Figure 1B), while the inhibitory effect was higher than 80% after 48 (Figure 1C) and 72 h (Figure 1D). The combination treatment with vemurafenib did not significantly alter the effects of the inhibitor.

To analyze whether CrataBL is also toxic to healthy cells, elevated concentrations of CrataBL were tested in human foreskin fibroblasts (HFF-1) cells. After 24 (Figure 1E) and 48 h (Figure 1F), no effects were observed in cell viability, even in combination with vemurafenib. However, the highest concentration (200 µM) of both treatments, alone and with the chemotherapy, reduced cell viability (approximately 40%) after 72 h (Figure 1G). It is important to emphasize that CrataBL affected more than 60% of SK-MEL-28 cell viability with 100 µM after 72 h, indicating the specificity of CrataBL in impairing the viability of SK-MEL-28 cells.

Concerning the mimetic peptides of CrataBL, Supplementary Figure S3 shows no significant differences in the effects of the treatment with peptides 26 and 27 alone or in combination with vemurafenib after 24, 48, and 72 h.

In summary, the results indicate that rBbKIm initially decreased melanoma cell viability but displayed a subsequent reversal. CrataBL exhibited potent inhibitory effects on melanoma cell viability, including a selective impact on SK-MEL-28 cells. The combination with vemurafenib did not alter the inhibitory effects significantly. The mimetic peptides of CrataBL showed consistent effects on cell viability, both alone and in combination with vemurafenib, over the tested time frame.

### 2.2. CrataBL Inhibits the Proliferation of SK-MEL-28

**Figure 2:**
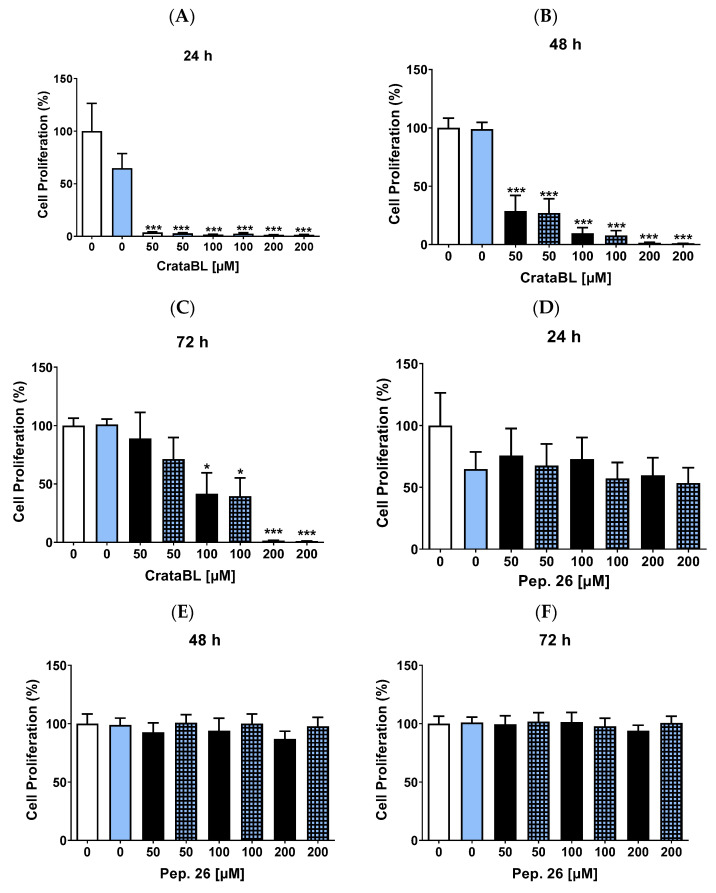

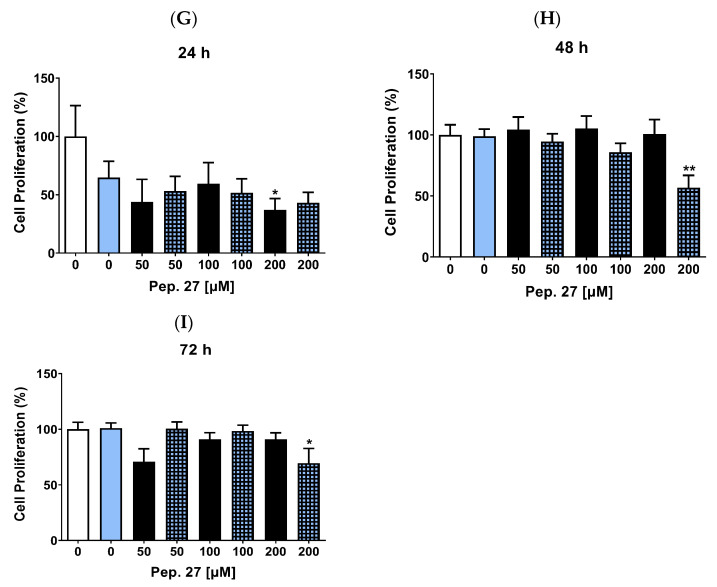
CrataBL inhibits the proliferation of SK-MEL-28. Treatment with CrataBL from 50 to 200 µM and the combined therapy in SK-MEL-28 for 24 (**A**), 48 (**B**), and 72 h (**C**). Concentrations of 50, 100, and 200 µM of isolated Pep. 26 and 2 µM of vemurafenib in three periods studied (**D**–**F**). Isolated Pep. 27 and the combination with chemotherapy drug at 24 (**G**), 48 (**H**), and 72 h (**I**). The white column represents the control (cells without any treatment), the blue light column represents 2 µM of vemurafenib, the black columns are the cells treated with CrataBL or peptides, and the light blue square columns are the combined treatment. (*) results are statistically different from control (* *p* < 0.05, ** *p* < 0.005, and *** *p* < 0.0005), one-way ANOVA, and Tukey test.

Since CrataBL interfered with cell viability, we investigated whether this effect was due to a decrease in the number of proliferating cells. CrataBL at all concentrations inhibited cell proliferation by 80–90%, and those treated with vemurafenib showed a similar effect to that of the protein alone in 24 h (Figure 2A). Moreover, 50 µM CrataBL alone or in combination with 2 µM of the chemotherapy agent showed inhibition of 45–48%, and with 100 µM CrataBL, the inhibitory effect was greater, and with 200 µM, the rate of proliferation was almost non-existent in 48 h (Figure 2B). The strong antiproliferative activity of CrataBL was maintained, suggesting its efficacy and resistance to prolonged treatment (Figure 2C). The peptide derived from CrataBL, Peptide 26 (Pep. 26) and Peptide 27 (Pep. 27), were also investigated. Pep. 26 treatments did not show promising results, even in combination with vemurafenib (Figure 2D–F), whereas Pep. 27 showed higher inhibition after 24 h (Figure 2G), although this effect was reversed after 48 (Figure 2H) and 72 h (Figure 2I), only the 200 µM combination therapy maintained the reduction in cell proliferation.

Thus, CrataBL exhibited potent anti-proliferative activity, inhibiting cell proliferation by 80–90% across various concentrations and maintaining its efficacy even with prolonged treatment. Peptide 26 did not show significant effects, while Peptide 27 initially displayed inhibition but lost its effectiveness over time. These findings suggest the potential of CrataBL as an effective agent against cell proliferation in our experimental model.

### 2.3. CrataBL Inhibits Cell Migration

**Figure 3:**
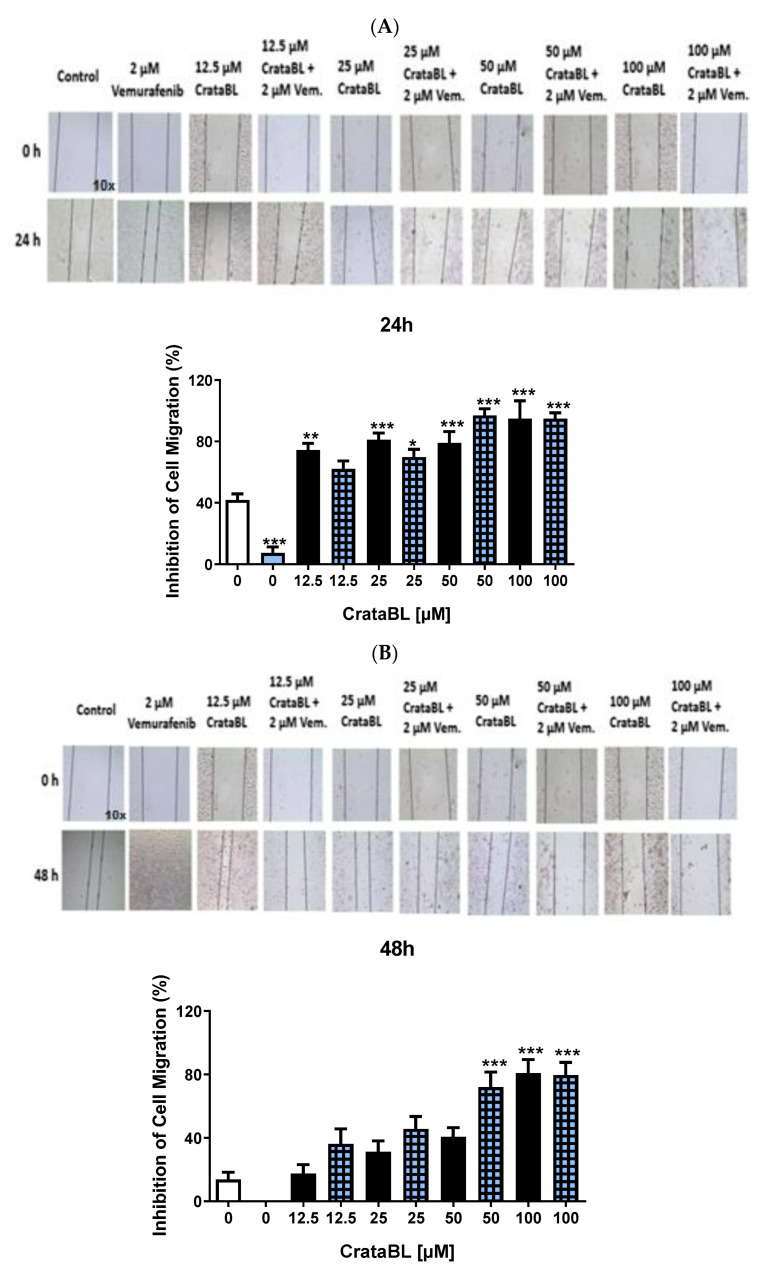

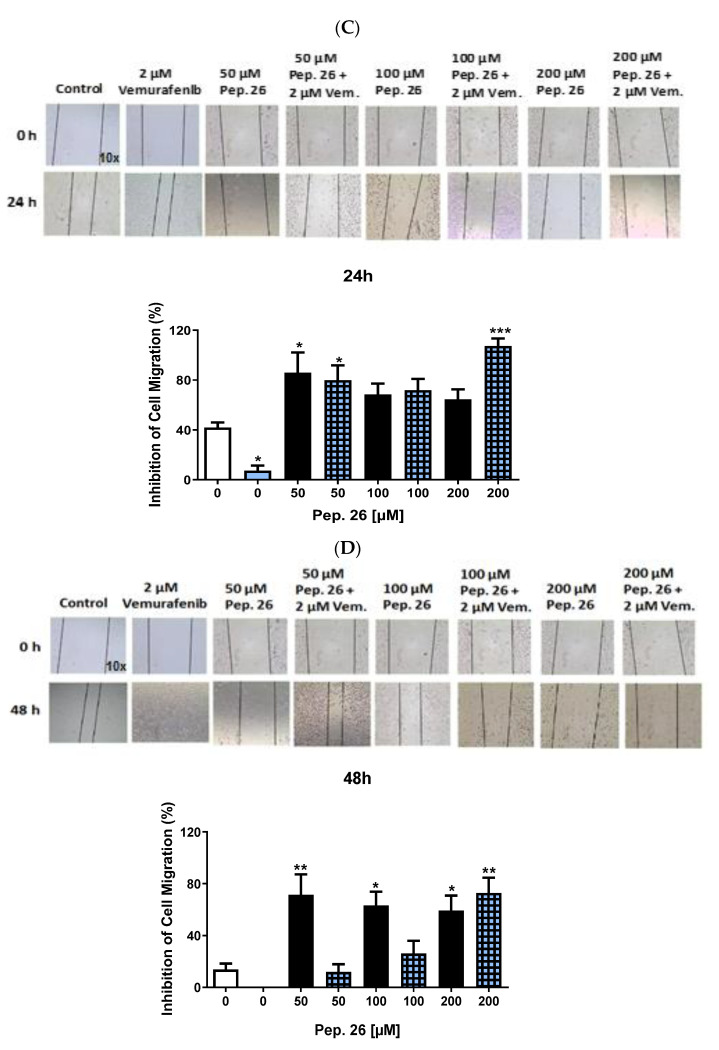

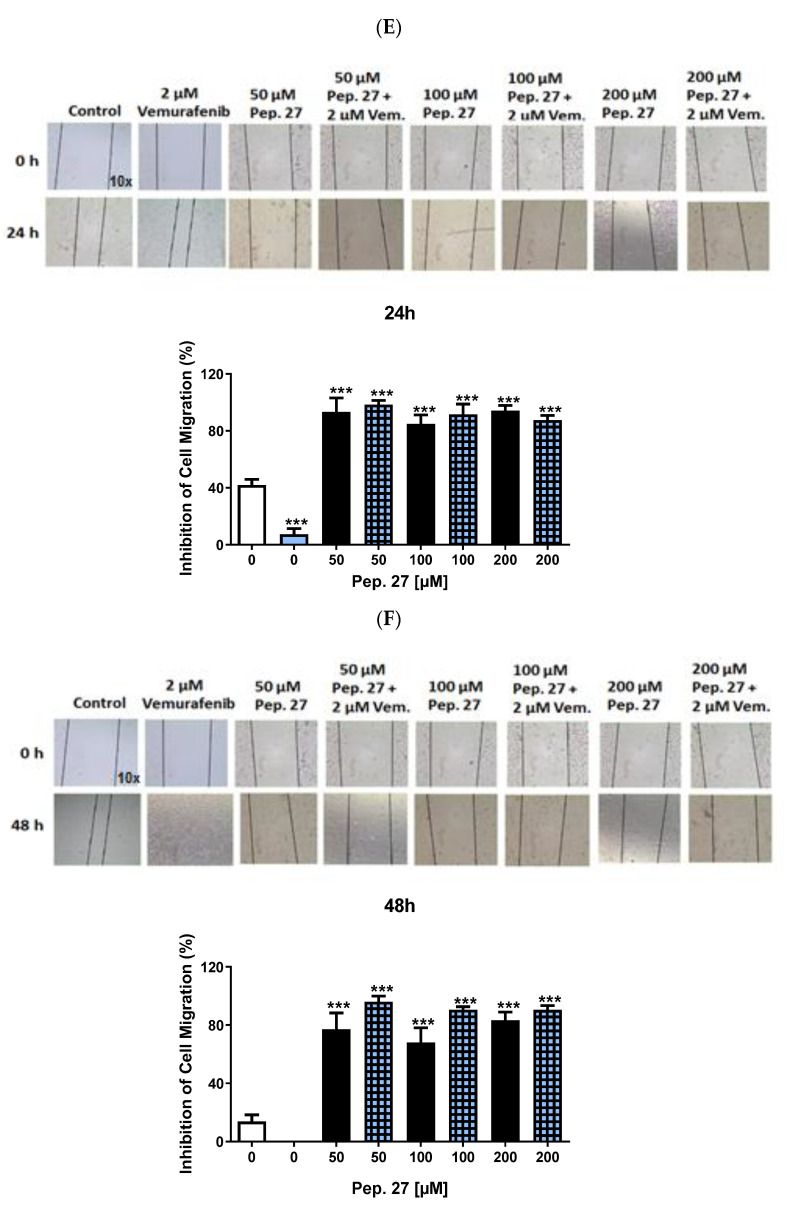
CrataBL and its derived peptides provoke inhibition in cell migration. Treatment with CrataBL from 12.5 to 100 µM and the combined therapy on SK-MEL-28 for 24 (**A**) and 48 (**B**) h. Concentrations of 50, 100, and 200 µM of isolated Pep. 26 and 2 µM of vemurafenib in two periods studied (**C**,**D**). Isolated Pep. 27 and the combination with chemotherapy drug at 24 (**E**) and 48 h (**F**). The white column represents the control (cells without any treatment), the blue light column represents 2 µM of vemurafenib, the black columns are the cells treated with CrataBL or peptides, and the light blue square columns are the combined treatment. (*) results are statistically different from control (* *p* < 0.05, ** *p* < 0.005, and *** *p* < 0.0005), one-way ANOVA, and Tukey test.

We investigated cell migration, which is an important event that mediates melanoma invasion and metastasis. Concentration–response inhibition (>50%) was observed with all concentrations of CrataBL (Figure 3A). It is important to note that the observed increase in the size of the gap when treated with 100 µM CrataBL does not necessarily indicate cell death alone. Although high concentrations of CrataBL, such as 100 µM, may promote apoptosis, it is essential to consider other factors that could contribute to the observed migration results and exclude the potential confounding effect of cell death alone. At 48 h (Figure 3B), the effects were more prominent at concentrations of 50 and 100 µM CrataBL. Although vemurafenib alone (2 µM) did not affect cell migration, an inhibition rate of approximately 100% was achieved with the combination of 200 µM Pep. 26 of CrataBL and 2 µM vemurafenib (Figure 3C) with a 20% reduction in the inhibition potency after 48 h of treatment (Figure 3D). Lower concentrations of Pep. 27 inhibited cell migration by more than 80%, and the combination with vemurafenib improved the effectiveness of the peptide (Figure 3E,F).

Our findings indicate that CrataBL exhibits concentration-dependent inhibition of cell migration, with notable effects observed at concentrations of 50 and 100 µM. The combination of CrataBL with vemurafenib enhanced the inhibitory effects, particularly in the case of Pep. 26 and Pep. 27. These results suggest the potential of CrataBL and its peptides as promising candidates for suppressing melanoma cell migration and highlight the synergistic effects when combined with vemurafenib.

### 2.4. CrataBL Reduces the Invasion of Melanoma Cells by More Than 50% within 24 h of Treatment

**Figure 4:**
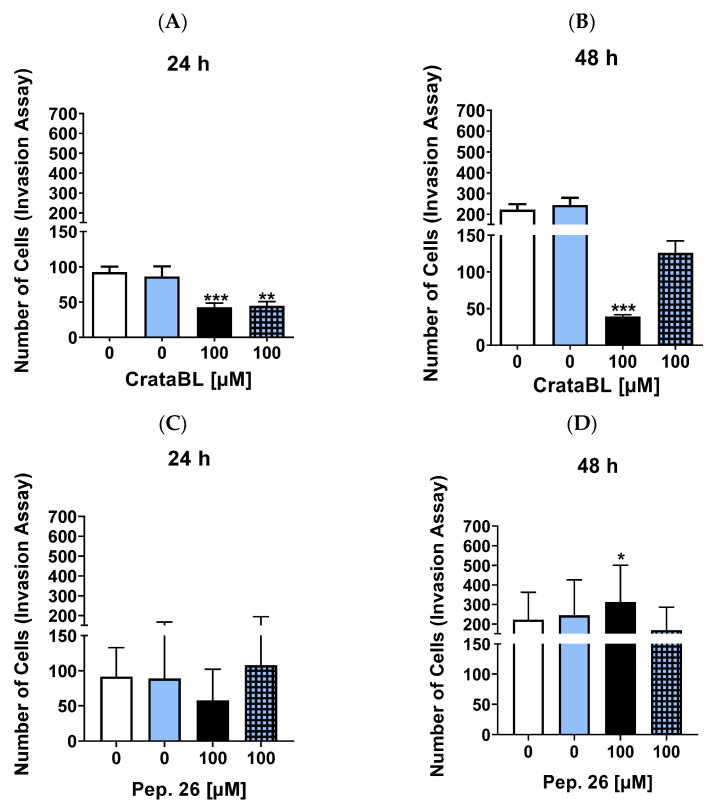

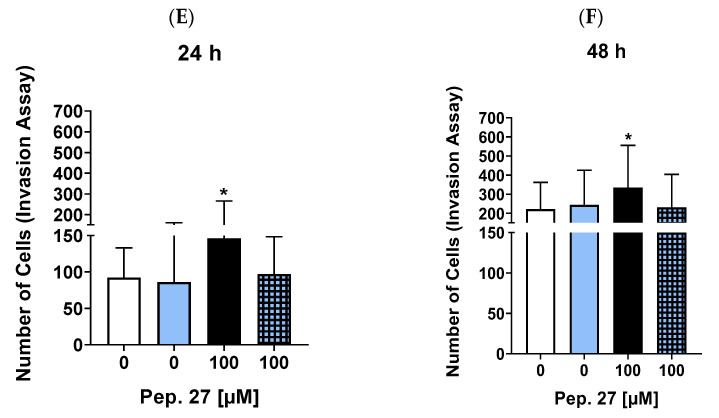
CrataBL decreased the invasive cells by more than 50% at 24 h of treatment. Invasion assay was performed with cells treated with 100 µM of CrataBL and combined therapy with 2 µM of vemurafenib in SK-MEL-28 for 24 (**A**) and 48 (**B**) h. Concentration of 100 µM of isolated Pep. 26 and with vemurafenib for 24 (**C**) and 48 (**D**) h. Isolated Pep. 27 and the combination with chemotherapy drugs in two periods studied (**E**,**F**). The white column represents the control (cells without any treatment), the blue light column represents 2 µM of vemurafenib, the black columns are the cells treated with CrataBL or peptides, and the light blue square columns are the combined treatment. (*) results are statistically different from control (* *p* < 0.05, ** *p* < 0.005, and *** *p* < 0.0005), one-way ANOVA, and Tukey test.

The cellular invasion has also been investigated as a mechanism that promotes metastasis in the late stages of various types of cancer. In Figure 4A, treatment with the protein both isolated and associated with 2 µM of vemurafenib significantly reduced the capacity of the cells to invade (50% in both). After 48 h of treatment (Figure 4B), the isolated protein showed a much better effect when not associated with chemotherapy, with a drastic inhibition of the cell invasion rate of more than 100%. Isolated Pep. 26 treatments showed a slight, but not statistically significant, decrease in invasion at 24 h (Figure 4C), and an increase in invasion at 48 h (Figure 4D). The same is true for Pep. 27 (Figure 4E,F), with an increase in the invasive profile, both after 24 and 48 h, leading to the conclusion that the protein itself was efficient in inhibiting the invasive capacity of cells, but the peptides were not even effective in combination therapy.

In conclusion, our results indicate that the CrataBL protein, both in isolation and in combination with vemurafenib, effectively reduced the invasive capacity of cells. However, the peptides derived from CrataBL, did not display significant inhibitory effects on cell invasion, and in some cases, they even increased the invasive profile. These findings highlight the superior efficacy of the protein itself in inhibiting cell invasion and raise questions about the effectiveness of the peptides in combination therapy.

### 2.5. CrataBL and Pep. 26 Reduced Tumor Cell Adhesion

**Figure 5:**
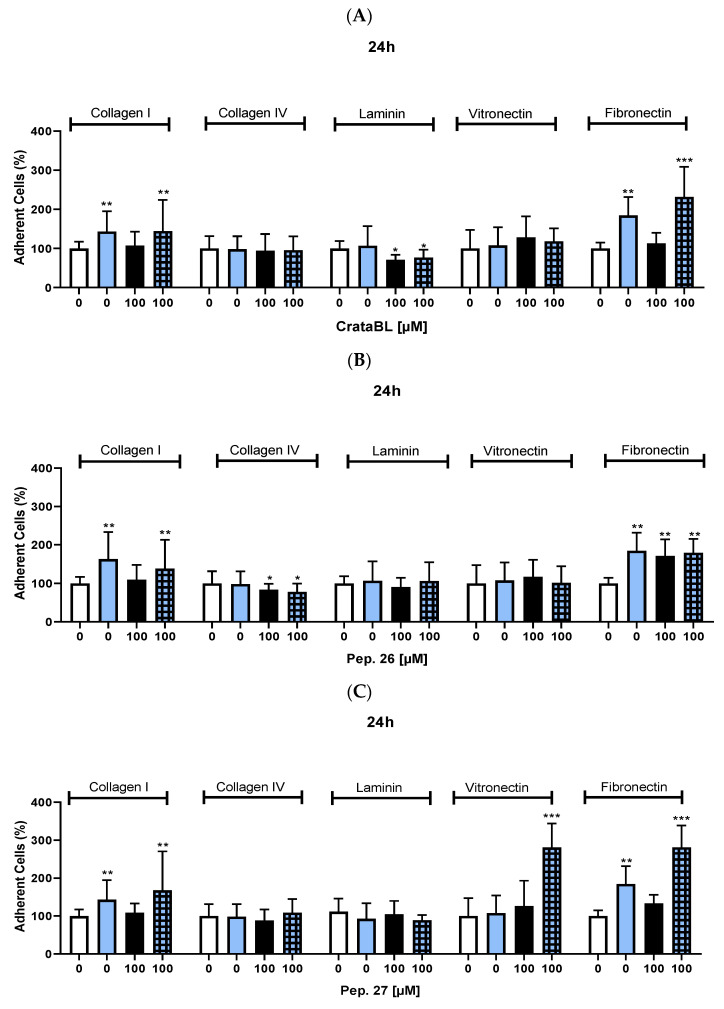
Effect of CrataBL alone and combination with vemurafenib on cell adhesion within 24 h. (**A**) Study of the effect of 2 µM of vemurafenib alone, 100 µM of CrataBL and their association on the number of adhered cells after 24 h of treatment. Treatment with Pep. 26 alone and together with chemotherapy in 24 h (**B**). Isolated and combined therapy with Pep. 27 (**C**). The white column represents the control (cells without any treatment), the blue light column represents 2 µM of vemurafenib, the black columns are the cells treated with CrataBL or peptides, and the light blue square columns are the combined treatment. (*) results are statistically different from control (* *p* < 0.05, ** *p* < 0.005, and *** *p* < 0.0005), one-way ANOVA, and Tukey test.

As these compounds interfere with cell migration and invasion, their effects on cell adhesion have been studied using the extracellular matrix (ECM) proteins: collagen I, collagen IV, laminin, vitronectin, and fibronectin. The study period was 24 h of interaction with the CrataBL protein or peptides at a concentration of 100 µM, 2 µM of chemotherapy alone, and in combined therapy. CrataBL treatment alone and in combination with the chemotherapy drug decreased the adhesion of SK-MEL-28 cells to laminin, whereas vemurafenib alone increased cell adhesion to collagen I and fibronectin (Figure 5A). In the treatment with Pep. 26, isolated or combined with vemurafenib, there was a slight decrease in adherence to collagen IV, while there was an increase in adherence to collagen I and fibronectin due to the effect of vemurafenib (Figure 5B), a behavior that was repeated in the combined treatment with Pep. 27, where an increase in the number of cells adhering to collagen I, vitronectin, and fibronectin was observed (Figure 5C).

In summary, the CrataBL protein and its peptides exhibited differential effects on cell adhesion to various extracellular matrix proteins. CrataBL treatment, either alone or in combination with chemotherapy, decreased cell adhesion to laminin. Vemurafenib alone increased cell adhesion to collagen I and fibronectin. The peptides, Pep. 26 and Pep. 27, showed complex effects on cell adhesion, with increases and decreases depending on the specific extracellular matrix protein and the presence of vemurafenib. These findings highlight the intricate interaction between these compounds and cell adhesion mechanisms.

### 2.6. CrataBL and Its Fragment Peptides Induce Apoptosis in Melanoma Cells

**Figure 6:**
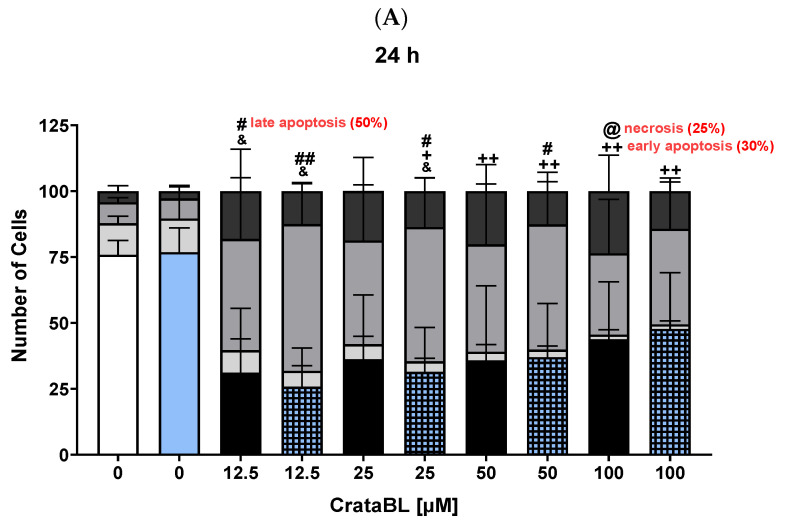

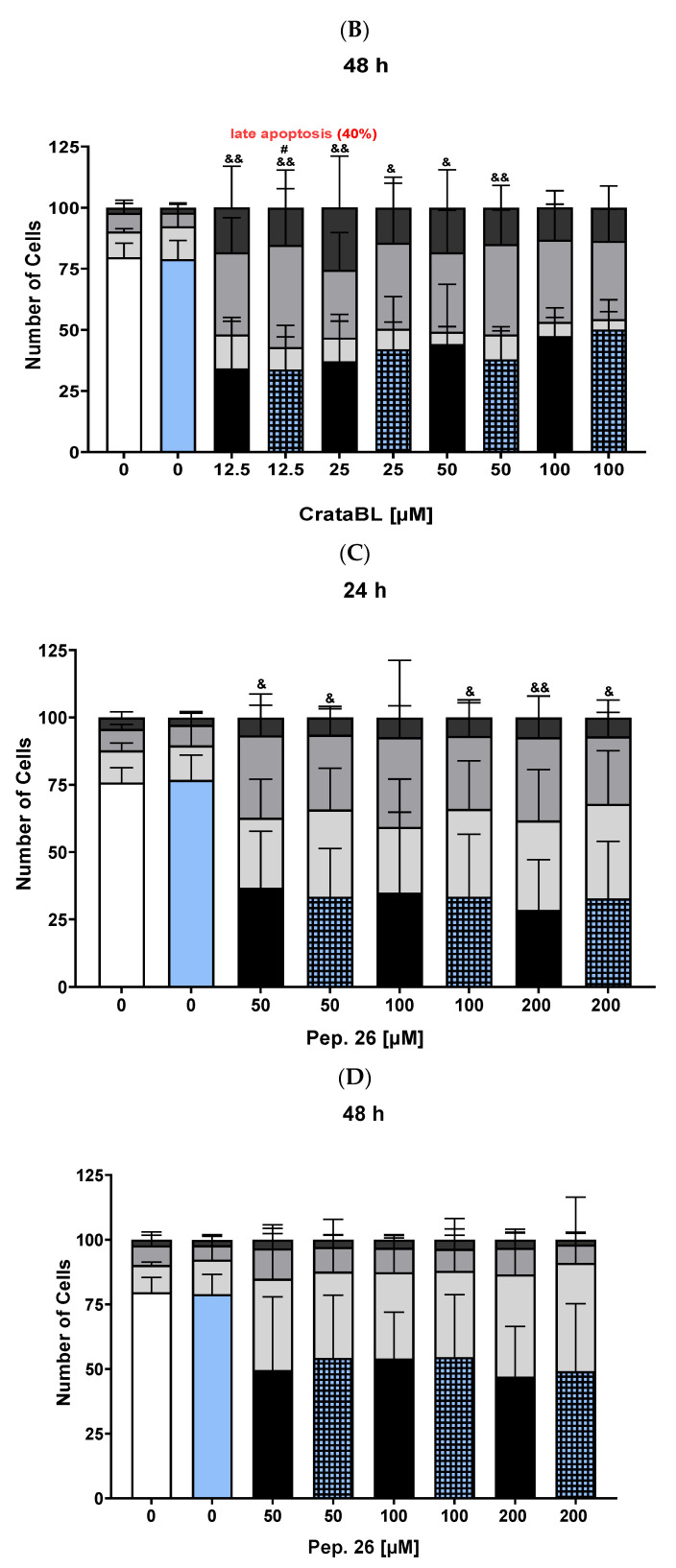

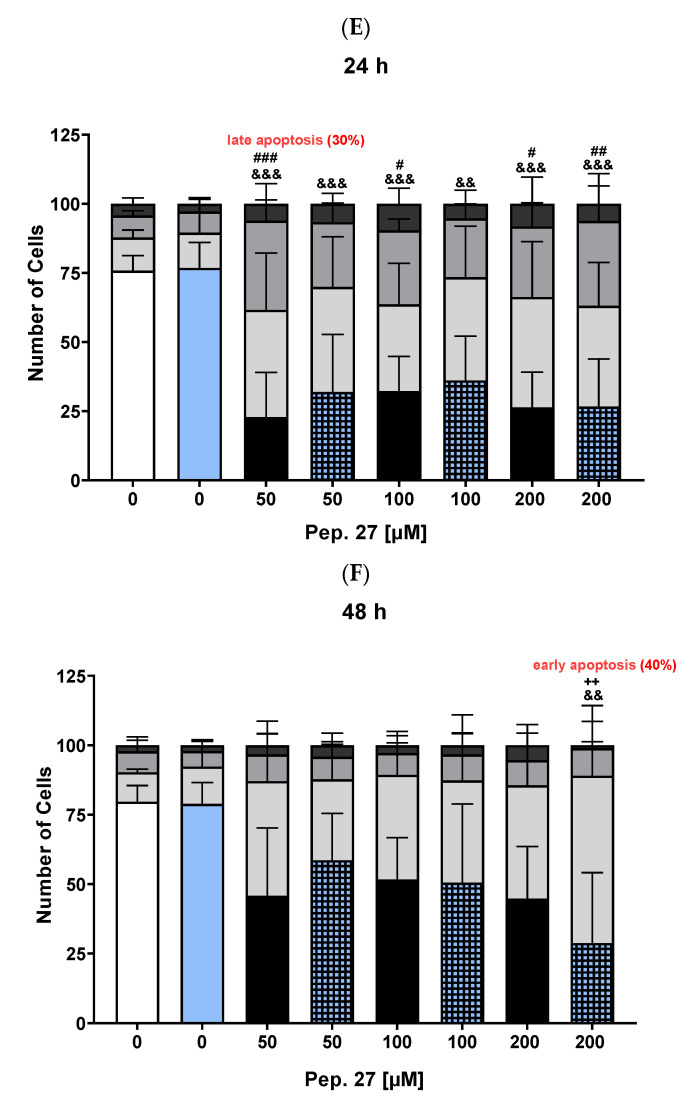
CrataBL and its fragment peptides induce apoptosis in melanoma cells. Treatment with CrataBL from 12.5 to 100 µM and the combined therapy in SK-MEL-28 for 24 (**A**) and 48 (**B**) h. Concentrations of 50, 100, and 200 µM of isolated Pep. 26 and with 2 µM of vemurafenib in two periods studied (**C**,**D**). Isolated Pep. 27 and the combination with chemotherapy drug at 24 (**E**) and 48 h (**F**). The white column represents the control (cells without treatment), the blue light column represents 2 µM of vemurafenib, black columns are the cells treated with CrataBL or peptides, and light blue square columns are the combined treatment viable cells; light gray for cells in early apoptosis stage (according to the treatment applied in each column); medium gray for cells at late apoptosis; and dark gray for cells in necrosis stage. (&) indicates that the viable cell results of the treated are statistically different from the control viable cells (& *p* < 0.05, && *p* < 0.005, and &&& *p* < 0.0005), (+) cell results in early apoptosis of the treated are statistically different from the control cells in the phase of early apoptosis (+ *p* < 0.05, and ++ *p* < 0.005), (#) indicates that the results of cells in late apoptosis of the treated are statistically different from the control of cells in late apoptosis (# *p* < 0.05, ## *p* < 0.005, and ### *p* < 0.0005), and (@) indicates that the results of cells in necrosis of the treated are statistically different from the control of cells in necrosis (@ *p* < 0.05), one-way ANOVA, and Tukey test.

Since CrataBL showed deleterious effects on the proliferation, migration, and invasion of SK-MEL-28 cells, and the peptides were promising for cell migration inhibition, the next step was to investigate the compounds involved in cell death. As shown in Figure 6A, after 24 h of treatment with the purified protein, an increase in the number of cells in late apoptosis was detected at the lowest concentration (50%): the combination with chemotherapy induced cells in the late phase of apoptosis, resulting in a significant increase. After 48 h (Figure 6B), the effect of the protein on the number of viable cells remained. With Pep. 26, after 24 h of treatment, there was a reduction in the number of viable cells (Figure 6C). Combined treatment did not alter the cell death profile, even after 48 h (Figure 6D). The results obtained using Pep. 27 (Figure 6E) showed a reduction in the number of viable cells at the initial concentrations and the induction of late apoptosis. After 48 h (Figure 6F), Pep. 27 maintained the reduction but with an increase in initial apoptosis in combination with chemotherapy (at 200 µM). In summary, CrataBL decreased the number of viable cells over time, whereas Pep. 27 at the highest dose and in combination with vemurafenib induced early apoptosis in treated cells (40%) after 48 h of treatment.

These data demonstrated that CrataBL exhibited a time-dependent decrease in the number of viable cells. Pep. 27, particularly at the highest dose and in combination with vemurafenib, induced early apoptosis in the treated cells, with a 40% reduction observed after 48 h of treatment. These findings highlight the impact of CrataBL and its peptides on cell viability and apoptosis, providing insights into their potential mechanisms of action.

### 2.7. CrataBL Reduces the Expression of Phosphorylated Src Protein and NF-kB 50 kDa

**Figure 7:**
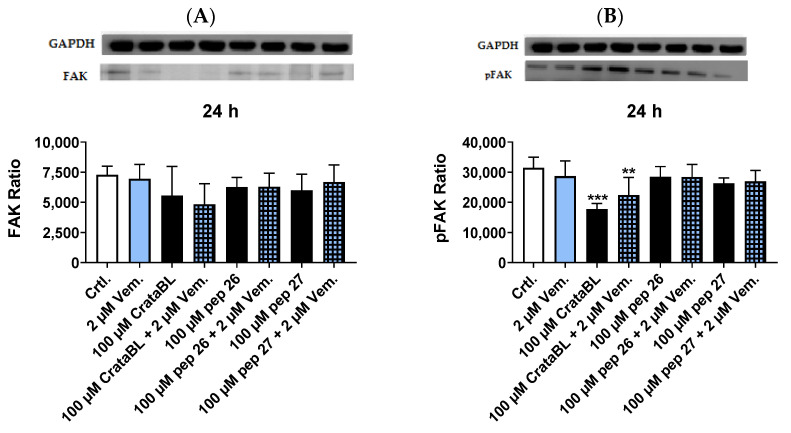

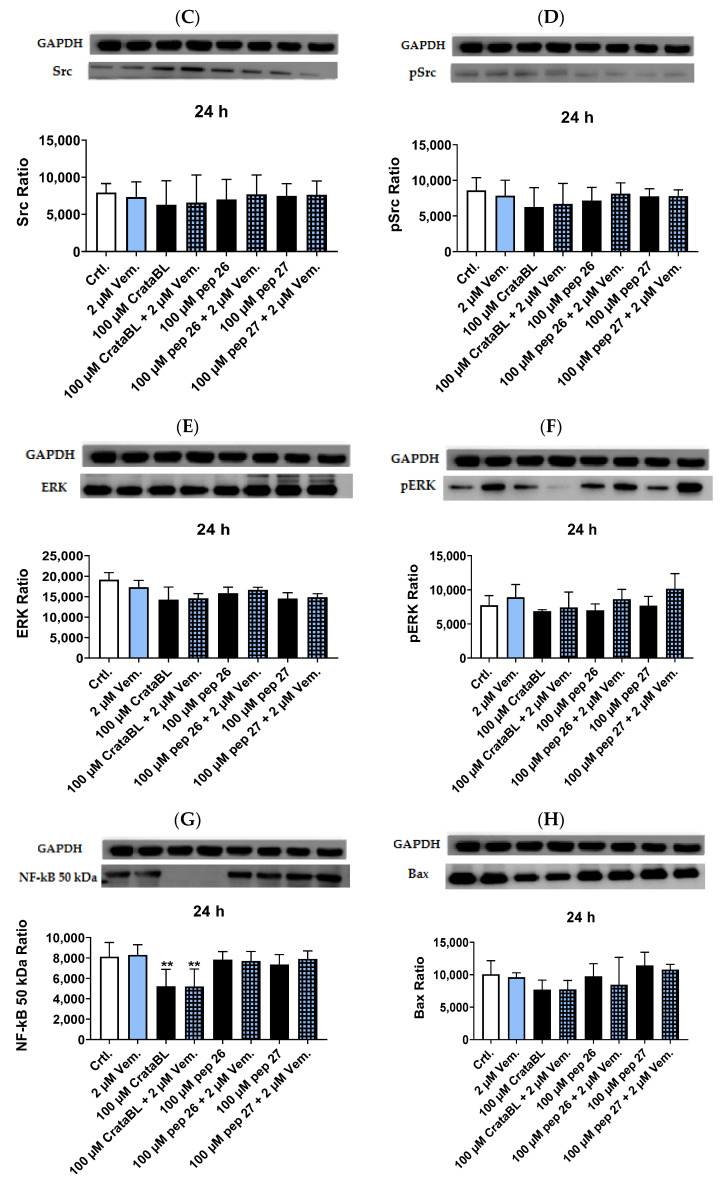
CrataBL reduces the expression of phosphorylated Src protein and NF-kB 50 kDa after 24 h of treatment. The Western blotting assay was performed with cells treated with only 2 µM of vemurafenib, 100 µM of CrataBL and the combination therapy, 100 µM of isolated Pep. 26 and with vemurafenib, and 100 µM isolated Pep. 27 and the combination with chemotherapy in SK-MEL-28 for 24 h measuring the ratio expression of protein FAK (**A**) and phosphorylated FAK (**B**). All these compounds were tested with the same conditions described before except measuring the expression of Src (**C**), pSrc (**D**), ERK (**E**), pERK (**F**), NF-kB 50 kDa (**G**), and Bax (**H**). The white column represents the control (cells without treatment), the blue light column represents 2 µM of vemurafenib, the black columns are the cells treated with CrataBL or peptides, and the light blue square columns are the combined treatment. (*) results are statistically different from control (** *p* < 0.005, and *** *p* < 0.0005), one-way ANOVA, and Tukey test.

To better understand the mechanisms of CrataBL and its peptides, Western blotting was performed to analyze some of the proteins implicated in the processes of proliferation, adhesion, invasion, inflammation, cell survival, and apoptosis [20,21,22]. In Figure 7A,B, levels of focal adhesion kinase (FAK) and phosphorylated FAK (pFAK) proteins are highlighted; CrataBL at 100 µM alone and in combination with 2 µM vemurafenib showed a slight decrease in FAK, although this was not statistically significant; however, CrataBL alone showed a reduction in more than 45% of pFAK, and the combined treatment also showed a reduction, even though not as great as the treatment alone, suggesting that the effect was more dependent on CrataBL. Peptides 26 and 27 and the combined treatment did not affect FAK and pFAK. Regarding proto-oncogene tyrosine-protein kinase Src (Src) (Figure 7C), phosphorylated Src (pSrc) (Figure 7D), extracellular signal-regulated kinase (ERK) (Figure 7E), and phosphorylated ERK (pERK) (Figure 7F), we observed that treatment with CrataBL, peptides, and vemurafenib alone and in combination with chemotherapy did not alter these signaling proteins, only in Figure 7F it is possible to state that vemurafenib caused a slight increase in pERK. Nuclear factor kappa B (NF-kB) 50 kDa and Bcl-2-associated X protein (Bax) are proteins capable of regulating cell death by apoptosis; although, results indicated that the compounds did not interfere with Bax protein expression (Figure 7H), CrataBL alone and combined with vemurafenib successfully decreased NF-kB 50 kDa levels (Figure 7G).

Overall, the Western blot analysis provided insights into the effects of CrataBL and its peptides on key signaling proteins involved in cell processes. While the treatment had limited effects on FAK, Src, ERK, and Bax, it demonstrated an impact on pFAK and NF-kB 50 kDa levels, indicating potential involvement in cellular pathways related to cell adhesion and apoptosis.

### 2.8. Anti-Inflammatory Properties of CrataBL and Related Peptides

**Figure 8:**
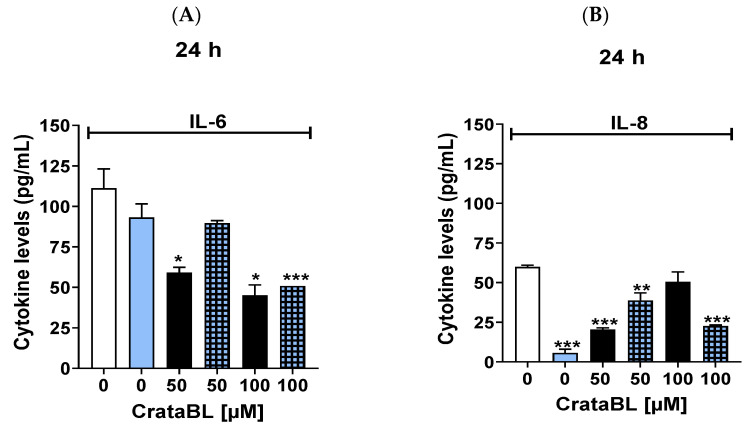

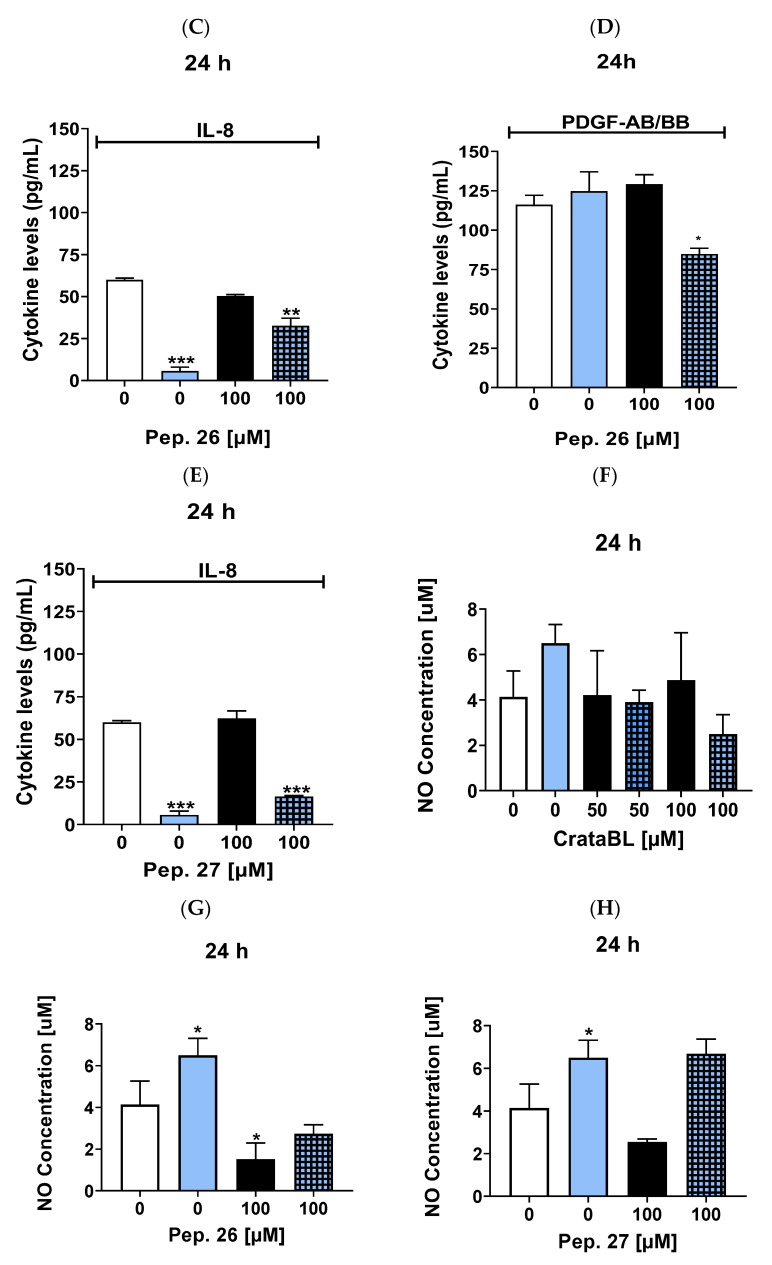
Anti-inflammatory properties of CrataBL and related peptides. Levels of interleukin 6 (**A**) and 8 (**B**) with treatment utilizing CrataBL of 50 and 100 µM and combined therapy for 24 h in SK-MEL-28. Concentrations of interleukin 8 and PDGF-AB/BB cytokine with treatment with Pep. 26 (**C**,**D**) and Pep. 27 (**E**) and in combination with vemurafenib. Concentrations of NO in SK-MEL-28 for 24 h using these components: CrataBL (**F**), Pep. 26 (**G**), and Pep. 27 (**H**), and the combined therapy. The white column represents the Control (cells without any treatment), the blue light column represents 2 µM of vemurafenib, the black columns are the cells treated with CrataBL or peptides, and the light blue square columns are the combined treatment. (*) results are statistically different from control (* *p* < 0.05, ** *p* < 0.005, and *** *p* < 0.0005), one-way ANOVA, and Tukey test.

Other components that participate in tumor development and are responsible for the signaling, activation, and regulation of cellular events include the release of nitric oxide (NO) and pro-inflammatory cytokines, which activate various oncogenic processes, such as cell invasion and proliferation in melanoma, and the ability to metastasize [23,24]. The anti-inflammatory properties of CrataBL and its related peptides were also evaluated using the pro-inflammatory cytokines interleukin 6 (IL-6), interleukin 8 (IL-8), and platelet-derived growth factor -AB, -BB (PDGF-AB/BB) (Figure 8A–E), and NO release (Figure 8F–H). A concentration of 100 µM of CrataBL reduced IL-6 level by more than 50% and showed no significant difference in IL-8. The combination (100 µM CrataBL and 2 µM vemurafenib) did not alter the CrataBL-IL-6 profile and showed a positive effect in decreasing IL-8, probably due to the properties of vemurafenib (Figure 8A,B).

The peptides (Figure 8C–E) did not modify the IL-8 and PDGF-AB/BB profiles as vemurafenib did, and combination therapy, in this case, reduced this pro-inflammatory cytokine. There was a slight increase in NO with CrataBL treatment (1.2-fold), in contrast to vemurafenib with which the NO concentration increased to 6.5 µM (increased 1.63-fold) (Figure 8F). There was a significant decrease in NO production at the concentration of 100 µM associated with vemurafenib. Peptide 26 (100 µM) significantly reduced NO production and neutralized the increase in the chemotherapy treatment alone (Figure 8G). Although Pep. 27 decreased the NO production in the isolated treatment (100 µM), the combination did not reverse the increase promoted with this chemotherapy agent (Figure 8H).

Overall, CrataBL demonstrated anti-inflammatory properties by lowering IL-6 levels and having a slight impact on NO production. The combination therapy with vemurafenib showed positive effects on IL-8 reduction and NO production inhibition. The peptides had limited effects on pro-inflammatory cytokines and NO production, with Pep. 26 showing a significant reduction in NO production and Pep. 27 not reversing the increase caused by chemotherapy.

## 3. Discussion

The high mortality rate in patients with late-detected melanoma indicates the need for more effective drugs to combat this disease. Proteins isolated from plants have very effective anticancer effects and provide alternative solutions for studies to better understand this disease. rBbKIm and CrataBL were the studied compounds, and both have demonstrated tumor cell inhibitory activity; CrataBL showed antitumor activity in glioblastoma cells [13], whereas rBbKIm showed effects on prostate cancer cells [14]. Herein, the data demonstrate that CrataBL, but not rBbKIm, has cytotoxic properties against SK-MEL-28 cells, suggesting the specificity of these proteins for different tumor types.

It is also important to evaluate the effects of these compounds on non-tumor cells as indicators of toxicity. Thus, while a high concentration of CrataBL (200 µM) reduces melanoma cell viability by more than 80%, the same concentration interferes with the viability of healthy fibroblast cells by about 40%. This is relevant information as it shows that the protein has a better and more selective inhibitory effect on human melanoma cells than on healthy human fibroblasts.

The fact that CrataBL caused a decrease in cell viability over a prolonged treatment period (72 h) indicated that possible fragments of the protein could be effective against this type of tumor, and highlighted the possibility of investigating smaller, synthetic peptides with less immunogenicity. We sought to examine isolated protein and peptide treatments as well as combination therapy, as resistance to chemotherapy is common and of concern, and combination treatment may reduce the concentration necessary for the effectiveness of the treatment by reducing resistance. CrataBL also reduced melanoma cell proliferation by more than 60% when applied at a concentration of 100 µM; mainly at a concentration of 200 µM the reduction was greater than 95%. It was also observed that at the lowest used concentration, 50 µM, and after 72 h, there was an increase in proliferation possibly due to the availability of amino acids provided by the ability of protein degradation and at this concentration the effect caused by amino acid delivery did not offset the deleterious effect on proliferation observed at higher concentrations. Hence, a greater supply of free amino acids for cell consumption increases metabolism and, consequently, cell viability and proliferation [25]. This effect was maintained even after 48 and 72 h, indicating that the efficacy was maintained over a long period of treatment. Peptides 26 (derived from the native protein), and 27 (retro inverse sequence of Pep. 26), did not show a significant decrease in cell proliferation, and chemotherapy did not alter the profile obtained with peptides in monotherapy. The data obtained thus far allow us to conclude that the CrataBL protein has better efficacy than the peptides and the chemotherapeutic agent itself, and that the addition of the chemotherapeutic agent to the treatments (with the protein and peptides) does not significantly contribute to the reduction in cellular proliferative activity.

An important event for the process of tumor invasion and metastasis is cell migration, and in this respect the results obtained with the protein and peptides were positively surprising as CrataBL inhibited cell migration by approximately 70% at the lowest used concentration, 12.5 µM, reaching approximately 100% at 100 µM. The evaluation of cell viability and time course experiments’ results provided additional insights into the effect following CrataBL treatment at the concentration below 100 µM. These results indicated that the observed effects on migration are not primarily driven by cell death alone, but rather other factors are at play. To specifically address the potential induction of apoptosis by high concentrations of CrataBL (such as 100 µM), we employed flow cytometry or immunoblotting to analyze markers associated with apoptosis. This analysis indicated that the observed increase in the gap size was not solely attributed to cell death (Figure 3A). Instead, it suggests that other mechanisms, such as impaired cell migration, contribute to the observed results of CrataBL treatment. In this regard, the efficacy of the protein was much higher than that of chemotherapy, as treatment with vemurafenib alone reduced cell migration only slightly (approximately 10%) compared with the control.

Careful analysis of combined therapy provides information that, at low protein concentrations, this association is detrimental because the interaction of CrataBL and vemurafenib prevented the protein from acting. However, with an increase in the concentration of CrataBL combined with chemotherapy, the inhibition of cell migration was increased or maintained, suggesting that chemotherapy may enhance the ability of CrataBL to inhibit cell migration, although it did not show a synergistic effect in the previous assays. Moreover, a concentration of 100 µM completely inhibited cell migration while under our experimental conditions, vemurafenib alone had no inhibitory effect on melanoma cell migration after 48 h of treatment.

These peptides effectively blocked the migratory behavior of the cells. Pep. 26 alone (after 24 h) reduced cell migration in a dose-dependent manner, and the most pronounced and promising effects were achieved in combination with vemurafenib, leading to a reduction of approximately 100%. Vemurafenib failed to reduce cell migration; however, the Pep. 26 and vemurafenib combination therapy was even more potent in reducing cell migration (approximately 80%) than CrataBL alone after 48 h of treatment. In the case of Pep. 27, its effect was much better than that of Pep. 26, with an inhibition percentage greater than 80%, and the combination with vemurafenib did not modify the peptide efficacy. The use of D-amino acids is a peptide modification strategy that aims at greater stability because D-amino acids are more resistant to proteolysis, thereby increasing their half-life [26]. CrataBL and Pep. 26 decreased the adhesion of SK-MEL-28 cells mediated by some extracellular matrix proteins that may contribute to the powerful effect of blocking migration and invasion.

Tissue homeostasis maintains a perfect balance between cell proliferation and death, which are closely related events. CrataBL treatment led to a subtle alteration in the number of cells undergoing necrosis (25%); however, in both treatments and more importantly in the combination treatment, there was a significant induction of cells to a state of late apoptosis. Both peptides decreased the number of viable cells after 24 h and Pep. 27 also induced late apoptosis; however, after 48 h, the efficacy of the peptides decreased noticeably with an increase in viable cells.

In cancer, many expression patterns are altered, leading to interference in the signaling pathways that contribute to cancer development and metastasis. For example, overexpression of ERK and NF-kB is associated with increased proliferation and decreased apoptosis [27,28]. Glycosaminoglycans are highly negatively charged elements that are free in the ECM, attached to the plasma membrane, and act as cell signaling modulators [29].

Owing to its high positive charge, CrataBL displays a strong binding affinity for glycosaminoglycans such as chondroitin sulfate type E and dermatan 4,6-O-disulfate [10]. Interference in cell signaling was demonstrated by a reduction in p-FAK by CrataBL alone or in combination with vemurafenib. Inhibition of Src or pSrc was not observed, but the activation of these proteins by a compensatory activation system cannot be excluded. Inhibition of the FAK/Src pathway by cell surface receptors with CrataBL confirmed the results described here, including the inhibition of cell proliferation, migration, invasion, and induction of apoptosis. Peptides 26 and 27 probably act via different mechanisms as they do not alter the expression of FAK, Src, ERK, or their phosphorylated forms. There was a slight increase in phosphorylated ERK protein after adjuvant treatment with vemurafenib. The effect of vemurafenib was previously described by Halaban [30].

The reduction in NF-kB by CrataBL is quite relevant because overexpression of NF-kB in cancer is associated with increased cell survival as it plays an important role in inflammation, immunity, proliferation, and apoptosis [31].

Bax induces apoptosis, and its low expression prolongs the survival of patients with metastatic melanoma undergoing chemotherapy [21,32]. The apoptotic Bax protein was not altered by the treatment with CrataBL or the peptides, either alone or in combination with chemotherapy, leading us to conclude that the mechanism involved in the effect of these compounds is independent of Bax and related to the reduction in NF-kB p50, causing apoptosis and interfering with tumor growth [33].

Inflammation plays an important role in tumor initiation, progression, angiogenesis, and metastasis, and cytokines play an important role in modulating the antitumor response or, in the case of chronic inflammation, inducing malignant cell transformation and expression of pro/anti-inflammatory cytokines and cytokine receptors [24,34]. The role of cytokines in neovascularization and invasiveness correlates with NO production [35]. The anti-inflammatory properties of CrataBL have been reported in an experimental model of chronic obstructive pulmonary disease, leading to a reduction in the nitric oxide control of the inflammatory response [36,37]. Our data demonstrated an anti-inflammatory action on tumor cells due to the association of CrataBL and its related peptides with vemurafenib because NO production was significantly reduced, as well as the inflammatory cytokines IL-6 (30%, 24 h), IL-8, and PDGF-AB/BB. The combination treatment did not alter the effects of the protein, whereas the increase in IL-6 production elicited by treatment alone was suppressed by combination therapy.

The reduction in IL-8 levels following treatment with chemotherapy alone was greater than 90%. This characteristic enhanced the results of the combined therapy because, although the protein reduced IL-8, the combined therapy with CrataBL showed better efficacy than the monotherapy. This profile was not observed with the peptides; therefore, the reduction in this interleukin after the peptide-vemurafenib therapy may be due to chemotherapy alone. Studies show that high interleukin 6, interleukin 8, and PDGF-AB/BB levels in melanoma are associated with poor patient survival [38,39]. The elevation of interleukin 6 over-activates the Janus kinase/signal transducer and activator of transcription (JAK/STAT3) signaling pathway, which is responsible for tumor proliferation, invasion, and metastasis, in addition to suppressing the antitumor immune response [40]. It is also implicated in the activation of NF-kB, a promoter gene that mediates anti-apoptotic responses [41] and shows resistance to several drugs, such as the BRAF inhibitor (vemurafenib) and immunotherapies [42]. Interleukin 8, seldom found in healthy cells, when present on the surface of tumor cells, activates the most diverse pathways related to tumor growth, angiogenesis, and metastasis of malignancy [43]. Platelet-derived growth factor (PDGF) belongs to a family of mitogens that stimulate cell division of pericytes, fibroblasts, smooth muscle cells, and glial cells in the brain and other cells [44], its isoforms, PDGF-AA and -BB, promote the stromal formation and tumor growth in melanoma [45]. Therefore, it is relevant the CrataBL effects in reducing the excessive activation of these cytokines.

It should also be emphasized that the lectin properties of CrataBL may contribute to its anti-inflammatory properties; i.e., CrataBL binding to glycosaminoglycans removes this component from the extracellular environment, which can potentiate the activities of proteases, especially cathepsins [46,47] and kallikreins [48]. Thus, when CrataBL binds to sulfated glycosaminoglycans in addition to interfering with the activity of kallikrein 7, which is important in melanoma [49], it may interfere with the activities of extracellular proteases which are indirectly inhibited by protecting the enzymes from glycosaminoglycans that are attached to the cell surface, impairing the potential of these compounds to enhance the activity of proteases. Although kinin generation was not investigated in this study, several authors have shown that kinin receptors have important implications for cancer, such as increasing vascular permeability and stimulating cell proliferation, migration, and angiogenesis. They also play important roles in cancer growth and metastasis by activating inflammatory pathways (for a review see Costa [50]).

In conclusion, regarding all cellular event outcomes, a possible mechanism of action of CrataBL involves its capacity to bind to highly expressed matrix components at the tumor site, decreasing the adhesion of melanoma cells to laminin, directly or indirectly inhibiting the activity of various proteases (such as cathepsins and kallikrein 7) in the tumor cells, and blocking some key signaling proteins that decrease the proliferation, migration, invasion, and survival of SK-MEL-28 cells.

The fragment peptide (Pep. 26) affected cell migration and reduced the number of viable cells after 24 h of treatment. In addition, Pep. 27 was more effective in inhibiting cell migration and inducing late apoptosis during the first treatment period. Combination with chemotherapy, although not synergistically, improved the inhibitory capacity of CrataBL and the peptides on cell migration and caused a significant decrease in IL-6, IL-8, and PDGF-AB/BB cytokines.

This study highlights that such compounds may have multiple mechanisms of action, providing a perspective for research exploring the biochemistry of the interactions involved in these inhibitions.

## 4. Materials and Methods

### 4.1. Cell Culture

Kindly provided by Prof. Dr. Helena Bonciani Nader, Human foreskin fibroblasts (HFF-1) were cultured in Dulbecco′s Modified Eagle′s Medium (DMEM) High medium (Sigma-Aldrich, St. Louis, MO, USA) supplemented with fetal bovine serum (FBS; 15%) and Human melanoma cell line (SK-MEL-28), obtained from Prof. Dr. Gustavo Pereira (Unifesp), were cultured in DMEM High medium (Sigma-Aldrich, St. Louis, MO, USA) supplemented with 10% FBS. Both media contained penicillin/streptomycin (1%) and were incubated at 37 °C in a humidified atmosphere containing 5% CO_2_.

### 4.2. CrataBL Purification

As described by Araújo [8], the bark extract of *Crataeva tapia* was resuspended in saline solution (10%, *w*/*v*) and precipitated with ammonium sulfate. The 30–60% fraction containing the protein was dialyzed and subjected to SP-cellulose ion exchange chromatography and molecular exclusion chromatography using an AKTA system with 75 a Superdex column (Äkta purifier system; GE Healthcare Life Sciences, Amersham, UK). The eluate was dialyzed, measured using the Lowry method (1951) [51], and concentrated using a Speed Vac (Hetovac VR-1; Lab HETO Equipment, Allerød, Denmark) for application in cellular experiments. Protein purity was confirmed using Matrix-Assisted Laser Desorption Ionization—Time of Flight (MALDI-TOF).

### 4.3. Synthetic Peptides Derived from the Protein CrataBL

The CrataBL-derived peptide (Ac-Q-N-S-S-L-K-V-V-P-L-NH_2_) was named Pep. 26, which was synthesized based on the CrataBL reactive site region and Pep. 27 (Ac-L-P-V-V-K-L-S-S-N-Q-NH_2_) was the inverse of Pep. 26 with D-amino acids, resulting in Pep. 26 and Pep. 27 enantiomers. They were synthesized by Watson Bio (Houston, TX, USA), and their homogeneity, purity, and concentration were evaluated using reversed-phase high-performance liquid chromatography.

### 4.4. Cell Viability

The inhibitory effect was assessed using the Prestoblue reagent (InvitrogenTM, Thermo Fischer Scientific, Eugene, OR, USA) [52]. Cells were seeded into individual 96-well microplates (Corning, New York, NY, USA): HFF-1 and SK-MEL-28 (5 × 10^4^/well) and incubated for 24 h. Increasing concentrations of rBbKIm (5, 25, 50 and 100 μM) or CrataBL (5, 25, 50, 100 and 200 μM) in DMEM High medium with 10% (for SK-MEL-28 cells) and 15% (for HFF-1 cells) of FBS and in combination with Vemurafenib (2 μM) were used to treat cells for 24 h, 48 h and 72 h. After the period, 10 μL/well of Prestoblue was added, the plate was incubated for 10 min at 37 °C, and absorbance was measured at 560–590 nm. The experiment was performed in triplicate and was repeated at least three times.

### 4.5. Cell Proliferation

Bromodeoxyuridine (BrdU) Kit (Sigma-Aldrich, Mannheim, BW, Germany) was used to detect the synthesis of new Deoxyribonucleic acid (DNA) sequences [53]. SK-MEL-28 cells (5 × 10^4^) were seeded in 96-well microplates (Corning, NY, USA), and incubated for 24 h. Different concentrations (50, 10, and 200 µM) of CrataBL and protein-derived peptide have been used in combination with chemotherapy to treat for different periods (24, 48, and 72 h). Plates were incubated with BrdU reagent for 24 h at 37 °C, the medium was removed, and the denaturation solution was added for 30 min. The denaturing solution was discarded, and an anti-BrDU antibody (Sigma-Aldrich, Mannheim, BW, Germany) with a dilution of 1:1000 was added for 1 h and 30 min. The wells were washed several times and the substrate was added for chemiluminescence emission analyzed at 405 nm using a FlexStation Multi-Mode Microplate Reader spectrophotometer (Molecular Devices, San Jose, CA, USA). This experiment was performed in triplicate and was repeated at least three times.

### 4.6. Cell Migration

In 24-well plates, 1 × 10^5^/mL of SK-MEL-28 cells were seeded and cultured at 37 °C, 5% CO_2_ for 24 h. After reaching 90–100% confluence, 10 µg/mL of mitomycin (Sigma-Aldrich, St. Louis, MO, USA) was added 15 min before scraping. Using a P200, a risk was created in the center of the well, and a picture of the initial moment was taken (t = 0 h) with a camera (Sony Cyber-Shot) coupled to an inverted optical microscope. The medium was removed and the cells were treated with the respective drugs: 12.5, 25, 50, and 100 µM of CrataBL; 50, 100, and 200 µM pep. 26; 50, 100, and 200 µM pep. 27; and 2 µM of vemurafenib in each treatment. After 24 h, new photos were taken, as well as after 48 h. The size of the gap was quantified using the Image J program (ij153-win-java8), and graphs were plotted using GraphPad Prism 7 program [54]. The experiment was performed at least thrice.

### 4.7. Cell Invasion

In 24-well plates, Boyden Transwell/ThinCertsTM chambers (Greiner Bio-One, Frickenhausen, Germany) were used. Matrigel at a 1:6 dilution (in medium without FBS) was added to each insert and allowed to polymerize. After this period, 5 × 10^4^ cells/250 µL and 100 µM of each treatment associated with 2 µM of vemurafenib dissolved in culture medium without FBS were added to the top of the formed matrigel. Inside each well, 400 µL of medium containing 10% SBF was added and incubated for 24 and 48 h. Cells that remained inside the insert were removed with a cotton swab and cells that invaded located in the external region were fixed with 4% formaldehyde (*v*/*v*). After 30 min, the wells were washed with phosphate-buffered saline (PBS) and stained with 4′,6-diamidino-2-phenylindole (DAPI,10 µg/mL) for 15 min under slow shaking. The membranes were washed with PBS and the inserts were photographed with a camera connected to an inverted optical microscope (DMi 8, Leica, France). The stained nuclei of the cells were counted using the ImageJ program (ij153-win-java8), and graphs were plotted using the GraphPad Prism 7 program [55].

### 4.8. Cell Adhesion Assay

Adhesion molecules such as collagen I (8 µg/well), collagen IV (4 µg/well), laminin (4 µg/well), vitronectin (0.2 µg/well), and fibronectin (4 µg/well) were added (final volume of 100 µL/well at the defined concentrations described above) in 96-well plates and placed at 4 °C for polymerization for 24 h. After this time, 100 µL/well of 1% bovine serum albumin (BSA) dissolved in PBS was added as a control substrate and incubated for 1 h at 37 °C. BSA was removed and plates were washed with PBS and 5 × 10^6^ cells/well and treatment compounds (100 µM CrataBL, peptide 26, peptide 27, and the conjugation with 2 µM vemurafenib) resuspended in DMEM High medium containing 10% FBS were added to the wells. After 4 h of incubation at 37 °C, the supernatant was removed, carefully so as not to touch the bottom of the wells, washed with PBS, and fixed with 4% paraformaldehyde for 1 h at 4 °C. Subsequently, the fixative was removed and the wells were washed again with PBS and stained overnight with toluidine blue. Excess dye was removed and the wells were washed with PBS. One hundred microliters of 1% sodium dodecyl sulfate (SDS) was added, and after 30 min, the fluorescence was measured at 540 nm using a Spectra Max Plus 384 spectrophotometer (Molecular Devices, Atascadero, CA, USA).

### 4.9. Cell Death Assay

SK-MEL-28 cells were incubated in 24-well plates at a density of 5 × 10^4^ cells/well at 37 °C, 5% CO_2_ for 24 h. Subsequently, the culture medium was removed and cells were treated for 24 and 48 h with these compounds: 12.5, 25, 50, and 100 µM of CrataBL; 50, 100, and 200 µM pep. 26; 50, 100, and 200 µM pep. 27; and/or 2 µM vemurafenib for each treatment. After the treatment period, the supernatant was collected, the wells were washed with PBS, the cells detached with versene for 5 min, then the supplemented medium was added, and the Eppendorf tubes were centrifuged at 3500 rpm, for 5 min, at 25 °C. Cells were resuspended in a binding buffer containing 1.25 µL Annexin V/FITC (cellular fluorescein isothiocyanate) (30 µg/mL) and 1.25 µL Propidium Iodide (50 µg/mL) using the BD Pharmigen Kit (BD Biosciences, San Jose, CA, USA). The incubation period was 10 min and readings were performed using a flow cytometer (BD Accuri C6, San Jose, CA, USA), with an analysis of at least 10,000 events [56].

### 4.10. Western Blotting

SK-MEL-28 (5 × 10^6^ cells) seeded in 6-well plates and incubated for 24 h. The culture medium was removed and concentrations of 100 µM of CrataBL, 100 µM of Pep. 26, 100 µM of Pep. 27, and 2 µM of vemurafenib in each treatment were added and its effect was evaluated after 24 h. To quantify proteins using micro bicinchoninic acid (microBCA) (Thermo Fisher Scientific, Rockford, IL, USA) the supernatants were collected and PBS containing 0.25 mM of sodium orthovanadate was added to the wells and successive scrapings were performed to detach the cells. The samples were centrifuged and the supernatants were discarded, this step was repeated and the precipitates were resuspended in lysis buffer containing a cocktail of protease inhibitors (Sigma, St. Louis, MO, USA) and phosphatases (0.25 mM sodium orthovanadate). The samples were disrupted using several freezing and heating steps. After centrifugation, supernatants were collected. Thirty micrograms of total protein were added to each well and the gels (15%) were run [57]. The gels were transferred to polyvinylidene fluoride membranes in which a blocking solution (5% BSA in 25 mM Tris buffer + 192 mM glycine and 0.1% (*v*/*v*) Tween 20) was added for 2 h, then the membranes were washed with TBST (buffer 25 mM Tris + 192 mM glycine and 0.1% (*v*/*v*) Tween 20) and then incubated with a primary antibody solution containing anti-FAK, Src, ERK, phosphorylated FAK, phosphorylated Src, phosphorylated ERK, NF-kB and Bax antibodies (BD Biosciences, San Jose, CA, USA) in with a dilution of 1:1000 in (5% BSA), 200 mM Tris buffer, pH 8, containing 0.15 M NaCl and 0.05% Tween 20, under gentle stirring at 4 °C overnight. The next day, the membranes were washed with TBST and a secondary antibody solution (rabbit IgG) (BD Biosciences, San Jose, CA, USA) with a dilution of 1:1000 (in 1% BSA (*w*/*v*) + 0.025 M Tris + 0.192 M glycine, and 0.1% (*v*/*v*) Tween 20) was added for 2 h and washed with TBS (without Tween 20). The membranes were then visualized using the photo documenter Bio-Rad/Bio-Imaging Systems after the addition of SuperSignal reagents (SuperSignal West Pico Chemiluminescent Substrate, Thermo Fisher Scientific, Carlsbad, CA, USA) [58] in a ratio of 1:1. The endogenous protein glyceraldehyde-3-phosphate dehydrogenase (GAPDH) was used as control to normalize the protein expression levels and Ponceau S reagent was used to ensure that the protein amounts were equalized and the transfers were properly executed. Images were acquired and quantified using ImageJ, and graphs were plotted using GraphPad Prism 7.

### 4.11. Cytokines Quantification

The cell culture method for this dosage was similar to that used to measure NO levels. In 24-well plates, 5 × 10^4^/mL SK-MEL-28 cells were seeded and cultured at 37 °C, 5% CO_2_ for 24 h; after confluence was reached, cells were treated with 50 µM and 100 µM of CrataBL; 100 µM pep. 26; 100 µM pep. 27, and 2 µM of vemurafenib in each treatment and incubated for another 24 h. Cellular secretome was collected (100 µL) in Eppendorf tubes and stored in a freezer at −80 °C for later use. Protein quantification of the samples was performed using a microBCA (Thermo Fisher Scientific, Rockford, IL, USA), and cytokine dosages were analyzed using Luminex 200 equipment (Thermo Fisher Scientific, Rockford, IL, USA), where the evaluated molecules were PDGF-AB/BB, IL6, and IL-8. Forty micrograms of total protein were used for this analysis [59].

### 4.12. Nitric Oxide Measurements

5 × 10^4^/mL SK-MEL-28 cells were plated in a 24-well plate for 24 h at 37 °C, 5% CO_2_. After this period, the cells were treated with 50 and 100 µM CrataBL, 100 µM pep. 26, 100 µM pep. 27, and 2 µM vemurafenib in each treatment and incubated for an additional 24 h. Cellular secretome was collected (100 µL) in Eppendorf tubes and immediately frozen at −80 °C for later use. The quantification of the samples was performed using microBCA (Thermo Fisher Scientific, Rockford, IL, USA) and 200 µg of total protein was used for NO dosages using the Nitric Oxide Analyzer (NOA^TM^ 208i-Sievers) in which chemiluminescence originates from the conversion of nitrite to nitric oxide [60] which is important in neovascularization.

### 4.13. Statistical Analysis

In the data analysis, the GraphPad Prism 7 program was used where the statistical differences were determined by the One-Way ANOVA method, the Tukey test (SigmaPlot 10.0), and the data with mean + standard deviation. Statistical differences were considered significant at * *p* < 0.05, ** *p* < 0.005, and *** *p* < 0.0005.

## Data Availability

Datasets used and/or analyzed during the current study are available from the corresponding author upon request.

## Supplementary Materials

The following supporting information can be downloaded at: https://www.mdpi.com/article/10.3390/ijms241310617/s1.
