# Supplementary material for: Impairment of SK-MEL-28 Development—A Human Melanoma Cell Line—By the Crataeva tapia Bark Lectin and Its Sequence-Derived Peptides
Source: Int J Mol Sci. 2023 Jun 25;24(13):10617. doi: 10.3390/ijms241310617 (PMC10342017; doi:10.3390/ijms241310617)
Supplement: Supplementary file 1 [file ijms-24-10617-s001.zip › ijms-2382629-supplementary.pdf]

International Journal of *Molecular Sciences*

MDPI

Supplementary Figures

Impairment of SK-MEL-28 Development—A Human Melanoma Cell Line—By the *Crataeva tapia* Bark Lectin and Its Sequence-Derived Peptides

Kathleen Chwen Ming Lie [1], Camila Ramalho Bonturi [1], Bruno Ramos Salu [1], Juliana Rodrigues de Oliveira [1], Márcia Bonini Galo [1], Patrícia Maria Guedes Paiva [2], Maria Tereza dos Santos Correia [2] and Maria Luiza Vilela Oliva [1,*]

[1] Department of Biochemistry, Universidade Federal de São Paulo, São Paulo 04044-020, Brazil; kalimryn@gmail.com (K.C.M.L.)
[2] Department of Biochemistry, Universidade Federal de Pernambuco, Recife 50670-910, Brazil
* Correspondence: mlvoliva@unifesp.br

Supplementary Figure S1

(A)

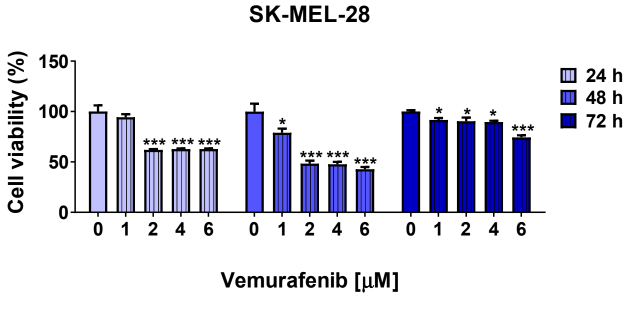

Supplementary Figure S1. Viability assay with different concentrations of Vemurafenib (chemotherapy drug used in melanoma SK-MEL-28) for 24, 48, and 72 hours. Zero represents the control (cells not treated), concentrations of 1, 2, 4 and 6 µM of vemurafenib tested in 24, 48 and 72 hours. (*) results are statistically different from control (* $p < 0.05$, ** $p < 0.005$ e *** $p < 0.0005$), one-way ANOVA and Tukey test.

Supplementary Figure S2

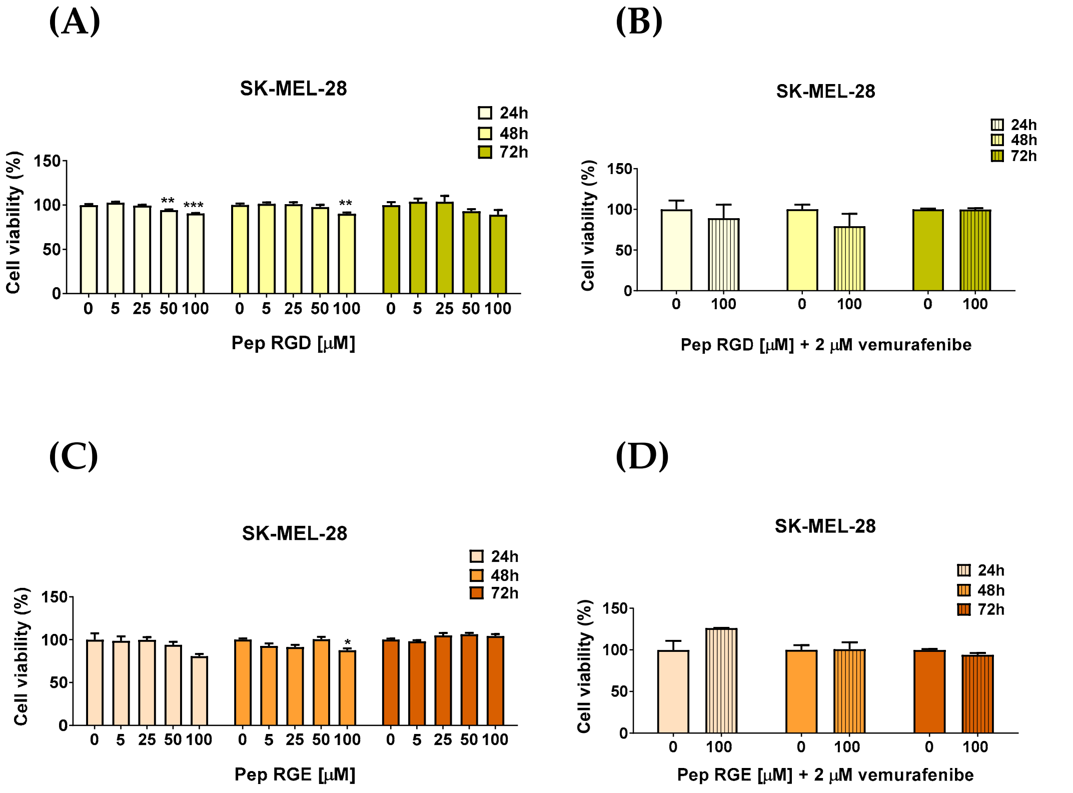

Supplementary Figure S2. Viability assay with increased doses of peptide RGD (**A**) and RGE (**C**), derived from protein rBbKIm, from 5 to 100 µM after 24, 48 and 72 hours of treatment. 100 µM of peptide RGD (**B**) and RGE (**D**) combined with 2 µM of vemurafenib for 24, 48, and 72 hours. The first column with number zero represents control (cells without any treatment) then increased doses of treatment with peptides and colours of yellor and orange getting darker means the prolong times of treatment. The columns with vertical lines are the combined treatment of 100 µM of peptides with 2 µM of vemurafenib in different periods. (*) results are statistically different from control (* $p < 0.05$, ** $p < 0.005$ e *** $p < 0.0005$), one-way ANOVA and Tukey test.

Supplementary Figure S3

(A)

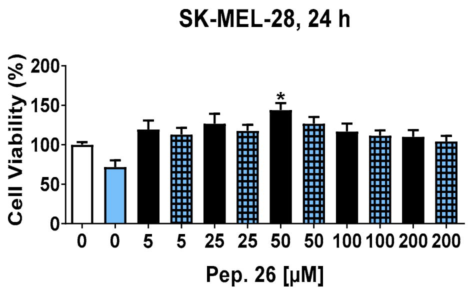

(B)

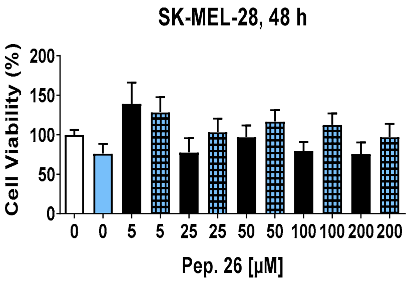

(C)

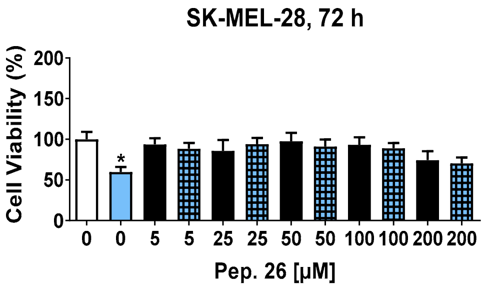

(D)

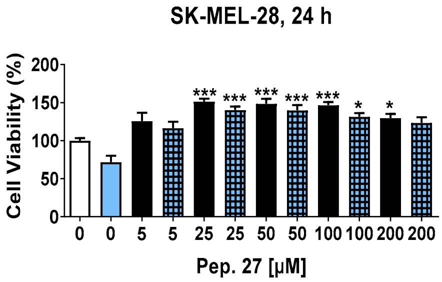

(E)

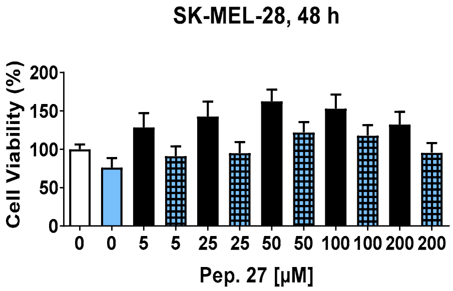

(F)

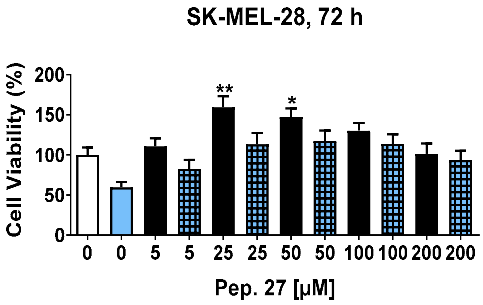

Supplementary Figure S3. Viability assay treated with 5, 25, 50, 100, and 200 µM of Pep. 26 derived from CrataBL and combined therapy with 2uM of vemurafenib in SK-MEL-

28 for 24 (**A**), 48 (**B**), and 72 (**C**) hours. The same concentrations were described above with Pep. 27 and with vemurafenib for 24 (**D**), 48 (**E**), and 72 (**F**) hours. The white column represents the control (cells without any treatment), the blue light column represents 2 μM of vemurafenib, the black columns are the cells treated with CrataBL or peptides and the light blue square columns are the combined treatment. (*) results are statistically different from control (* $p < 0.05$, ** $p < 0.005$, and *** $p < 0.0005$), one-way ANOVA and Tukey test.
